# Monte Carlo modeling of a Novalis TX Varian 6 MV with HD‐120 multileaf collimator

**DOI:** 10.1120/jacmp.v13i5.3960

**Published:** 2012-09-06

**Authors:** Luis Alberto Vazquez‐Quino, Brian Massingill, Chengyu Shi, Alonso Gutierrez, Carlos Esquivel, Tony Eng, Nikos Papanikolaou, Sotirios Stathakis

**Affiliations:** ^1^ Cancer Therapy and Research Center Department of Radiation Oncology University of Texas Health Science Center San Antonio TX USA; ^2^ Radiation Oncology Saint Vincent Medical Center CT USA

**Keywords:** Monte Carlo simulations, BEAMnrc, DOSXYZnrc, linac, Novalis TX, HD‐MLC

## Abstract

A Monte Carlo model of the Novalis Tx linear accelerator equipped with high‐definition multileaf collimator (HD‐120 HD‐MLC) was commissioned using ionization chamber measurements in water. All measurements in water were performed using a liquid filled ionization chamber. Film measurements were made using EDR2 film in solid water. Open rectangular fields defined by the jaws or the HD‐MLC were used for comparison against measurements. Furthermore, inter‐ and intraleaf leakage calculated by the Monte Carlo model was compared against film measurements. The statistical uncertainty of the Monte Carlo calculations was less than 1% for all simulations. Results for all regular field sizes show an excellent agreement with commissioning data (percent depth‐dose curves and profiles), well within 1% of difference in the relative dose and 1 mm distance to agreement. The computed leakage through HD‐MLCs shows good agreement with film measurements. The Monte Carlo model developed in this study accurately represents the new Novalis Tx Varian linac with HD‐MLC and can be used for reliable patient dose calculations.

PACS number: 87.10.Rt

## I. INTRODUCTION

Monte Carlo simulations represent potentially the most accurate method to for patient dose calculations in radiotherapy.^(1)^ The development of faster computational systems and the advancements of faster Monte Carlo simulation algorithms offer a unique opportunity for the use of Monte Carlo calculations in the clinical environment of radiation oncology. Many publications in the last few years have shown the potential of the Monte Carlo method for calculations of dose, especially for intensity‐modulated radiation therapy (IMRT)‐based plans.^1^, ^22^

The planning aspects of MLC‐based IMRT represent a challenge primarily because the IMRT beams consist of a large number of control points (small segments) which can suffer electronic disequilibrium.^23^, ^24^ For a complex intensity pattern, the dose distributions can be very sensitive to the detailed structure of the MLC.^23^, ^25^ It is evident from these reasons that there is a need for an accurate Monte Carlo model which can predict the accuracy of dose calculations in the presence of HD‐MLC. In order to accurately predict the dose deposition phenomena and achieve accurate dose calculations, the Monte Carlo model of the linac needs to be contrasted against direct measurements, such as ion chamber and film measurement, in a known geometry under standard conditions.

The high‐definition multi‐leaf collimator (HD‐MLC) on the Novalis TX system (Varian Medical Systems, Palo Alto, CA) has 60 leaf pairs. The inner 32 leaf pairs have a 2.5 mm width projection at isocenter, while the outer 28 leaf pairs have a projection of 5 mm at isocenter. The purpose of this study is to develop and benchmark a Monte Carlo model of the Novalis TX linear accelerator (linac) to be used for patient dose calculation. Monte Carlo dose simulations and direct measurements using both an ion chamber and EDR2 film of percent depth‐dose curves (PDDs) and dose profiles at different depths were performed. Finally, intra‐ and interleaf leakage of the HD‐MLC study was conducted, as well as a comparison of the energy fluence distribution and spectral distribution between fields defined by jaws and HD‐MLC.

## II. MATERIALS AND METHODS

Monte Carlo simulation, according to the American Association of Physicist in Medicine (AAPM) TG‐105, should be performed under the same conditions as the measurements.^(1)^ For this reason, all simulations present here were performed in a water phantom $\left(30\times 30\times 30{\;\text{cm}}^{3}\right)$ in order to meet the requirements for megavoltage photon dosimetry from AAPM report TG‐51.^(26)^

**Figure 1:**
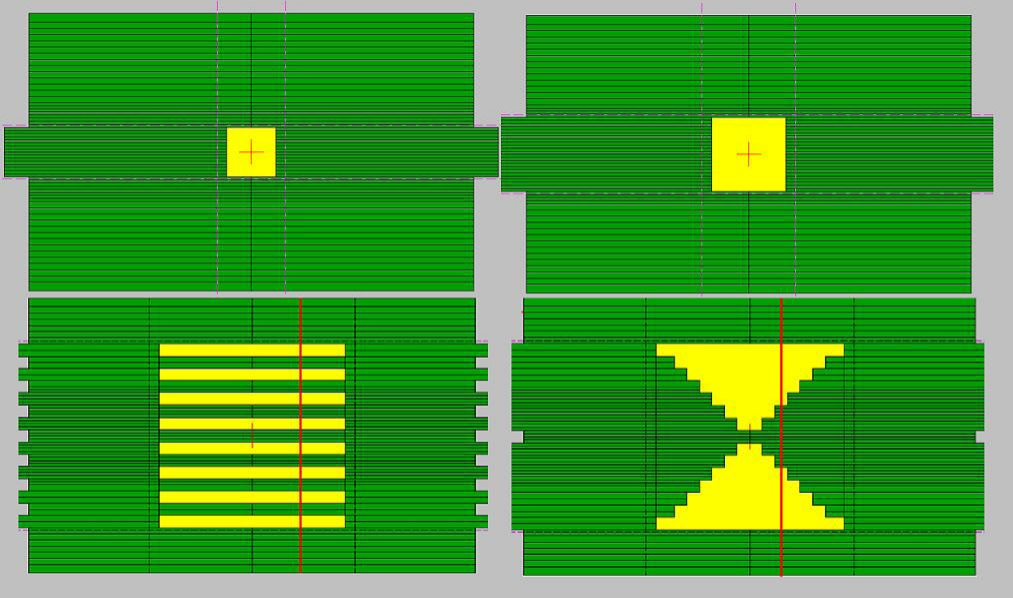
Different geometries created with HD‐MLC used for simulations.

A number of regular field sizes defined by jaws and HD‐MLC ranging from $1\times 1\;\text{to}\;20\times 20\;{\text{cm}}^{2}$ were used for the model of the linac. Also, sample irregular fields defined by the HD‐MLC were used to compare the results of the simulations with measurements. Furthermore, the intra‐ and interleaf leakage of the HD‐MLC was calculated using the Monte Carlo model of the linac and compared against measurements. All PDDs and profiles calculated at various depths using the Monte Carlo model were compared with commissioning data taken with a PTW liquid filled ionization chamber (microLion ionization chamber, 0.002cc; PTW, Freiburg, Germany).^27^, ^29^ Commissioning data were acquired in water tank with a step size of 5 mm and 2 mm for PDDs and profiles, respectively. All simulations and measurements were performed using the same geometric setup at 100 cm SSD. Profiles obtained at different depths in water were measured using water‐equivalent material and EDR2 film. Film calibration was performed to convert optical density into dose by irradiating different parts of the film to known doses ranging from 0 to 300 cGy at 5 cm depth in a solid water phantom. The film was scanned using a Vidar Advantage‐Pro (VIDAR Systems/Contex Group, Stockholm, Sweden) and RIT113 version 5.3 (Radiological Imaging Technology, Inc., Colorado Springs, CO) was used for analysis. The inter‐ and intraleaf leakage and leakage between the intersections of the leaf faces were also evaluated utilizing alternating leaf and closed field configurations, respectively. (see Fig. 1)

The Monte Carlo simulations of dose deposition were performed in two steps. First, a phase‐space file was obtained from the simulation of the treatment head at a location immediately after the HD‐MLC. The phase‐space files were then used as input for the dose calculations in the phantom.^3^, ^4^

First measurements were performed for open fields defined by jaws and then for open fields defined by the HD‐MLC with the jaws fixed at $20\times 20{\;\text{cm}}^{2}$. PDD curves were measured for open fields, and dose profiles at various depths were measured for both open and irregular fields using EDR2 film.

### A.1 BEAMnrc simulations

**Figure 2:**
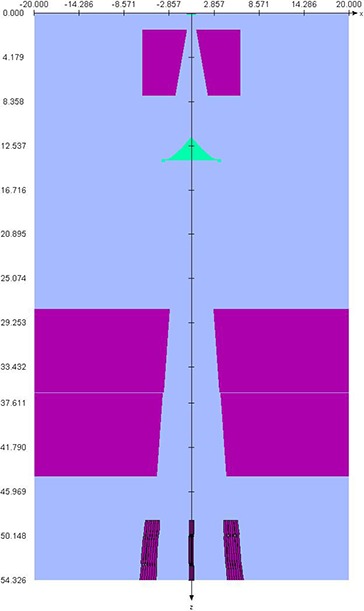
Novalis TX head in BEAMnrc.

Phase‐space files for the different field size of the Novalis TX 6 MV photon beams were created using the EGSnrc\BEAMnrc system.^2^, ^5^, ^12^ The cutoff energies used in the simulations were ECUT=700 KeV for electrons and PCUT=10KeV for photons. Monte Carlo simulations for monoenergetic beams ranging from 5.7 to 6.2 and FWHM varied from 0.110 to 0.140 cm were performed to find the best match with PDDs and profiles compared to measurements. Amonoenergetic source of kinetic energy of the beam of 6 MeV was used with full width at half maximum (FWHM) for the X and Y directions of 0.125 cm. Geometry and materials used to build the Monte Carlo model of the linear accelerator were based on machine specifications as provided by the manufacturer. The linac was structured in the following order: a target slab of tungsten and copper, primary collimator of tungsten, flattening filter, ion chamber, jaws (tungsten), and finally the option for Varian HD‐MLC (VARMLC) (Fig. 2). All materials used in the MC simulation were extracted from the 700 ICRU PEGS4 (preprocessor for Electron Gamma Shower) cross section data available in BEAMnrc, and met the specifications for the linac as provided by the manufacturer.

All simulations had a minimum requirement of 100,000 particles per ${\text{cm}}^{2}$ for each field; this was done to ensure reliable statistics in the phase space file generated by the BEAMnrc simulation. The phase‐space files were scored at a plane immediately after the jaws or immediately below the HD‐MLC, depending on definition of the field.

**Figure 3:**
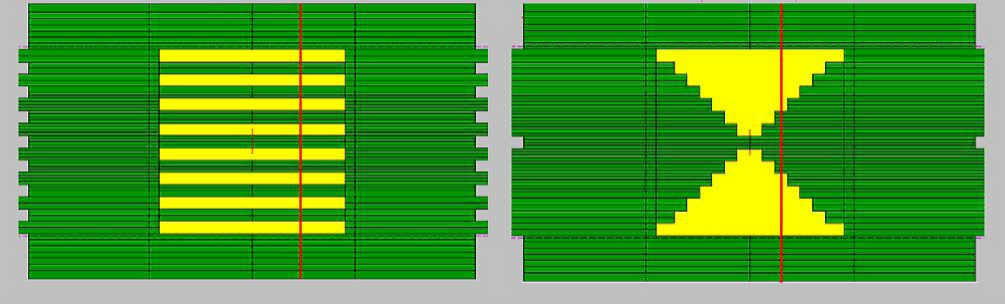
Irregular fields.

**Figure 4:**
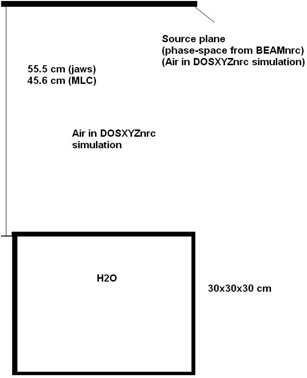
Scheme of phantom calculation in DOSXYZnrc.

The HD‐MLC defined fields were created using the Shaper version 7.0 (Varian Medical System, Inc., Palo Alto, CA). The HD‐MLC shapes were exported as text files and were used as input in the BEAMnrc input file for the linac. Four of the fields used in this study are presented in Fig. 2 —4×4 and $6\times 6{\;\text{cm}}^{2}$ fields and two arbitrary shapes used to investigate the dose calculation and modeling of the HD‐MLC for the Novalis TX Varian machine. Examples of irregular fields are shown in Fig. 3.

### A.2 DOSXYZnrc simulations

Monte Carlo dose calculations were performed using the phase space files described above as source input. The energy cutoffs in all the phantom simulations were ECUT=700 keV (rest mass+kinetic energy) for electrons and PCUT=10keV for photons. The energy thresholds for δ‐ray production (AE) and for photon production (AP) were set at 700 keV and 10 keV, respectively. ESTEPE, the maximum fractional energy loss per electron step, was set to 0.01 and the default parameters were used for the PRESTA (the Parameter Reduced Electron‐step Transport Algorithm) (ICRP 1991). PRESTA is an electron transport algorithm for use with electron Monte Carlo transport codes. PRESTA components are: a path‐length correction (PLC) algorithm which is based on the multiples scattering theory of Moliére and which takes into account lateral transport, and a boundary crossing algorithm (BCA) which ensures that electrons are transported accurately in the vicinity of interfaces.^(30)^ A statistical uncertainty (1σ) of less than 1% at $d_{\text{max}}$ has been achieved for most of the phantom dose calculations. A maximum statistical uncertainty of < 1.2% was accepted in the cases when uncertainties less than 1% were not achieved at $d_{\text{max}}$. (see Fig. 4.)

## III. RESULTS

### A.1 Square field simulations

**Figure 5:**
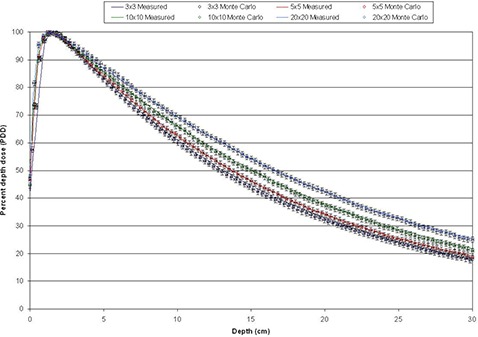
PDD obtained for fields defined by jaws.

Figure 5 shows the resulting PDDs obtained from the simulations for the fields defined by jaw settings. The solid lines represent the PDDs measured with ion chamber and the PDDs calculated by Monte Carlo simulation are presented as circles with error bars of 1% in the relative dose and 1 mm in depth. Similar results were obtained for PDDs obtained with 4×4, 6×6, 8×8, 12×12, and $15\times 15{\;\text{cm}}^{2}$ field sizes.

**Figure 6:**
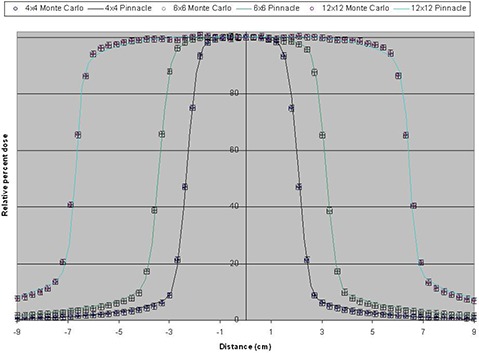
Profiles for fields defined by jaws.

Figure 6 shows examples of profiles obtained from the Monte Carlo simulation and compared with measured data with at 100 cm SSD and a depth of 10 cm in slabs of solid water equivalent.

**Figure 7:**
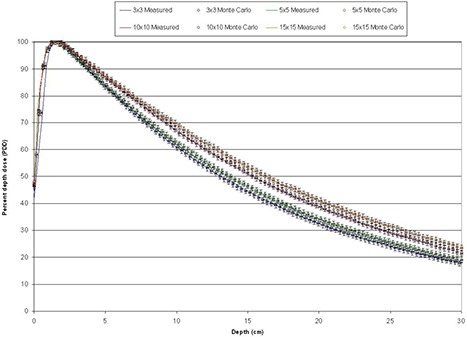
PDDs for fields defined by HD‐MLC.

Fields defined by the HD‐MLC with jaw settings at $15\times 15{\;\text{cm}}^{2}$ were calculated and resulting PDDs are presented in Fig. 7. Same criterion was used (1% and 1 mm) to analyze the PDDs obtained from HD‐MLC defined fields as in the jaws setting. PDDs for 3×3, 5×5, 10×10, and $15\times 15{\;\text{cm}}^{2}$ are presented. Solid lines represents measured PDDs at time of LINAC commissioning and circles with error bars Monte Carlo simulations.

**Figure 8:**
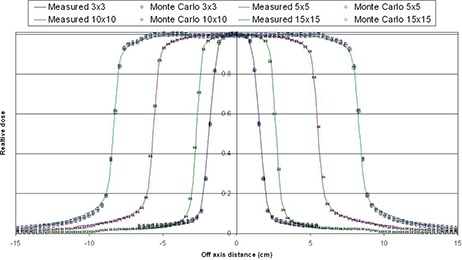
Profile comparison for HD‐MLC fields at 10 cm depth in water phantom.

Figure 8 shows the profile comparison for HD‐MLC defined field sizes for 3×3, 5×5, 10×10 and $15\times 15{\;\text{cm}}^{2}$ at 10 cm depth. Same criterion as before, and similar results within 1% and 1 mm were observed with the rest of the other field sizes simulated and compared. All fields were obtained for different depth (5, 10 and 20 cm) in the water phantom and compared with measured profiles; similar results were obtained with same criterion.

**Figure 9:**
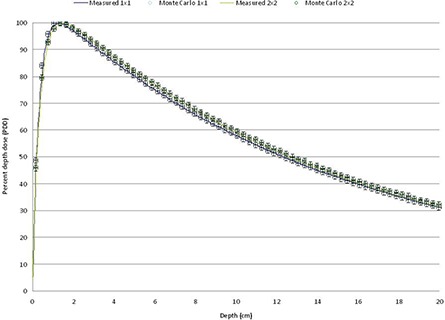
PDD of a 1×1 and $2\times 2{\;\text{cm}}^{2}$ field defined by HD‐MLC.

**Figure 10:**
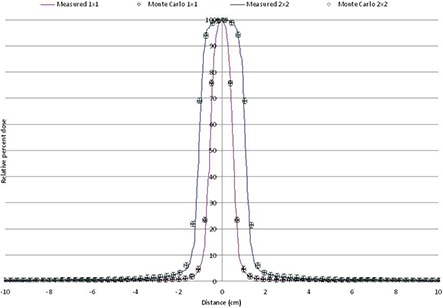
Profiles of a 1×1 and $2\times 2{\;\text{cm}}^{2}$ field defined by HD‐MLC at 7.5 cm depth in water phantom.

Special interest in the small field dosimetry was studied because of the capabilities of the linac under study with the HD‐120 HD‐MLC. Figure 9 shows PDDs for 1×1 and $2\times 2{\;\text{cm}}^{2}$ defined by HD‐MLC. And Fig. 10 shows profiles for 1×1 and $2\times 2{\;\text{cm}}^{2}$ fields defined by HD‐MLC.

### A.2 Irregular fields

**Figure 11:**
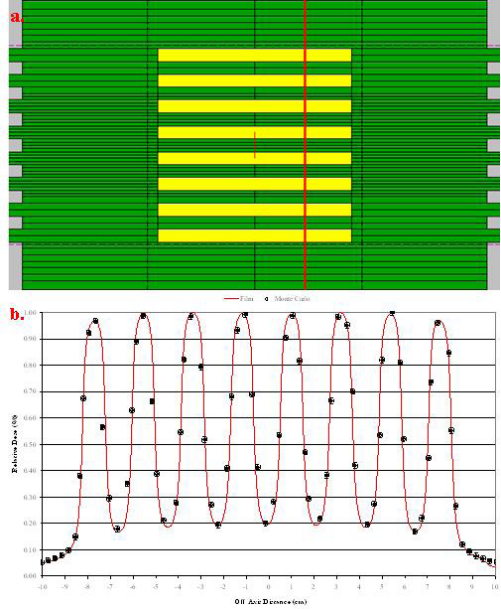
Irregular field and cross‐profile comparison.

**Figure 12:**
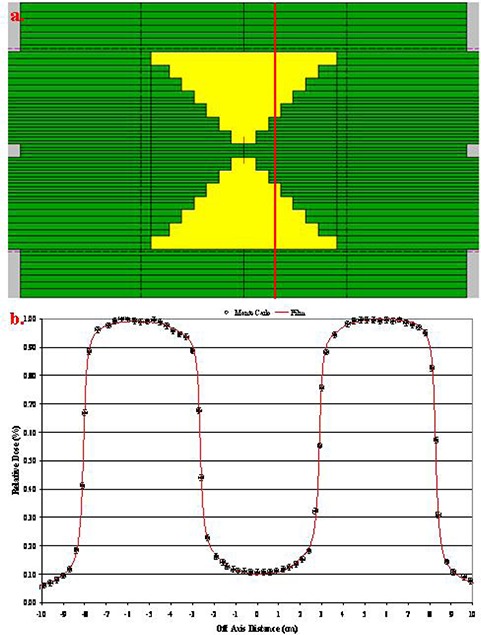
Irregular field and cross‐profile comparison.

Irregular fields were simulated ((Figs. 1c)and (1d). Profile comparisons between calculated and measured data using film at depth of 5 cm in solid water are shown in Figs. 11 and 12. The agreement between the measurements and calculations are within 1% and 1 mm and lies within the statistical parameters of our Monte Carlo simulations.

#### B. Leakage

**Figure 13:**
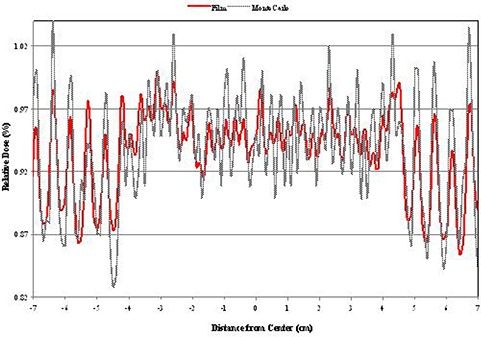
Leakage between the intersection of leaf faces.

A qualitative agreement was observed when evaluating the leakage between the leaf faces. The “peaks” expected at the intersection of the leaves and “valleys” expected through the central portion of the leaves occurred at the expected locations for both the film and model. The off‐axis accuracy was within ±1.0mm; however, the agreement between the measured and modeled relative percent dose deposition varied from <1% to ±7%. Figure 13 illustrates the results of evaluating the leakage between the leaf faces. Again, both BEAMnrc and DOSXYZnrc simulations were calculated so that they yield less that 1% statistical uncertainty for the Monte Carlo simulations.

#### C. Secondary electron production

**Figure 14:**
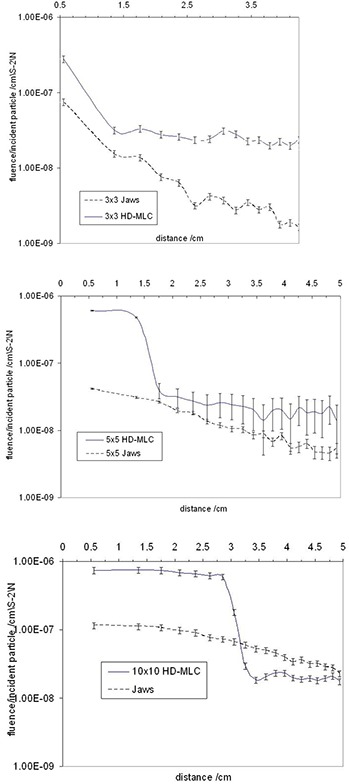
Secondary electron fluence analysis comparison.

The secondary electron production was found to be increased due to the presence of the HD‐MLC ((Fig. 14a) – (c)). The phase space files of the 3×3, 5×5, and $10\times 10{\;\text{cm}}^{2}$ fields were collected and compared for the same field sizes with and without the mMLC. The fluence of electrons generated when the HD‐MLC was attached was higher compared to the electron fluence without the HD‐MLC. Calculations showed increasing secondary electrons contribution from the HD‐MLC of approximately 4 to 6 times.

## IV. DISCUSSION & CONCLUSION

A Monte Carlo model of a Novalis TX machine with HD‐120 HD‐MLCs has been tested and benchmarked to produce phase‐space files to be used in future research in order to test the feasibility of clinical application of Monte Carlo simulation for calculation of dose in phantom and patient CT.

Comparison of data from a Novalis TX machine equipped with HD‐120 HD‐MLC with Monte Carlo simulations of regular and irregular fields has been shown, with a very good agreement between the Monte Carlo simulation and data from the commissioning of the linac and film dosimetry. Very good agreement is observed among the PDDs and profiles at depth between Monte Carlo simulations and data obtained from direct measurements taken using the linac for fields defined by jaws and HD‐MLC. The agreement is within 1% in the relative dose and 1 mm distance to agreement for all the regular field sizes investigated in this study (3×3, 4×4, 5×5, 6×6, 8×8, 10×10, 12×12, 15×15, and $20\times 20{\;\text{cm}}^{2}$). It is only in the buildup region of the 3times3 cm2 field PDD is where we observe deviation from the 1% and 1 mm but within 2%/2 mm for 1 or 2 points. Such deviations are most likely due to inaccuracies in the measurements due to setup uncertainties of the ionization chamber, leveling of the ionization chamber, and water tank and simplifications of the simulation components for increased efficiency. Nonetheless, the 2%/2 mm deviation is very well within acceptable criteria, especially since the behavior of the PDDs after $d_{\text{max}}$ is in good agreement with measurements.

The use of photon splitting combined with particle recycling has been shown in past studies performed by Kawrakow and Walters^(2)^ resulting in higher efficiency of the simulation, but our study observed a better accuracy in dose calculation with the use of recycling particles from the phase‐space in DOSXYZnrc simulation than a combination of both methods mentioned above.

The agreement of our Monte Carlo model was further validated by simulations of irregular fields. The calculations and measurements were in good agreement. The model was able to predict the dose to the peaks and valleys of the irregular fields very accurately. Furthermore, dose calculations for fields down to 1×1, 2×2, and $3\times 3{\;\text{cm}}^{2}$ were presented, and very good agreement was observed enabling the capabilities of our model for future IMRT and SRS applications.

Leakage between leaf faces in the closed position through the HD‐MLC is in the order of 1.0% to 7.0%. Discrepancies between the simulation and measurements for the leakage ranged from < 1 to ±7%. These deviations were assumed to be due to the fact that the film was only exposed to a low dose and the response in this range is very sensitive to calibration. The addition of the HD‐MLC component increases the electron contamination when compared against the fields defined by the jaws. This increase is in the order of 4 to 6 times. The dose to the secondary electrons can be significant, especially in the buildup region but this investigation was out of the scope of this study.

In conclusion, a Monte Carlo model of a Novalis TX machine with HD‐120 HD‐MLC has been tested and benchmarked to produce phase‐space files to be used in future research in order to test the feasibility of clinical application of Monte Carlo simulation for calculation of dose in phantom and patient CT.
